# Long-Term Cryopreservation May Cause Genomic Instability and the Premature Senescence of Cells

**DOI:** 10.3390/ijms25031467

**Published:** 2024-01-25

**Authors:** Mariia Shorokhova, Natalia Pugovkina, Victoria Zemelko, Olga Lyublinskaya, Tatiana Grinchuk

**Affiliations:** Department of Intracellular Signaling and Transport, Institute of Cytology, Russian Academy of Sciences, Tikhoretskii pr. 4, St. Petersburg 194064, Russia; natalia.pugovkina@gmail.com (N.P.); vzemelko@mail.ru (V.Z.); o.lyublinskaya@mail.ru (O.L.); grintat@bk.ru (T.G.)

**Keywords:** cryopreservation, genomic instability, karyotype, chromosomes, transformed Chinese hamster lung fibroblasts, endometrial mesenchymal stem cells, induced premature senescence

## Abstract

Cryopreservation is an essential step for utilizing various cell types for biological research and medical purposes. At the same time, there is a lack of data on the effect of cryopreservation, especially when prolonged, on the karyotype of cells. In the present work, we analyzed the genetic stability of cells subjected to a cryopreservation procedure. The objects were immortalized Chinese hamster lung fibroblasts (CHL V-79 RJK line) and human endometrial mesenchymal stem/stromal cells (eMSCs). We showed that short-term cryopreservation in liquid nitrogen for up to 6 months did not affect the karyotype stability of CHL V-79 RJK and eMSCs. On the contrary, karyotyping of G-banded metaphase chromosomes in cells underwent 10-year cryopreservation, which revealed genomic instability in both cell lines associated with the variability of chromosome number in cells, random chromosomal rearrangements, and condensation disorder in homologs. In addition, we found out that long-term cryopreservation of eMSCs does not affect the expression of their typical surface markers and morphology, but results in a significant reduction in proliferative potential and early manifestation of cellular senescence features upon eMSCs culturing. Thus, we concluded that the long-term cryopreservation of cells of different types and biological origin can lead to irreversible changes of their karyotype and acceleration of cellular senescence.

## 1. Introduction

Nowadays, long-term cryopreservation of human cells in liquid nitrogen for cell culture banks is considered a necessary tool for the development of regenerative medicine and related biotechnology fields. There are two strategies of cryopreservation: slow freezing and ultra-fast freezing (vitrification). Slow freezing is the most common, since it allows the use of lower levels of cryoprotectants. Thus, it promotes higher survival rates of the cells that experience cryopreservation for a long time and allows researchers and clinicians to expect that these cells sustain their genetic stability and physiological properties after thawing.

Despite the importance of this issue, the information about the possible impact of cryopreservation on cellular functions is sparse and ambiguous [1]. On the one hand, it is widely believed that cryopreservation, in particular short-term cryopreservation, from several days to several weeks, does not change, and even improves the phenotypic and genotypic stability of cells [2,3,4,5,6,7]. However, according to other studies, changes can occur in thawed cells that affect their intracellular structures and their genetic apparatus due to the cryopreservation process [1,8,9,10,11].

There are many factors that can affect cells during cryopreservation. There are data demonstrating that the use of cryoprotectants [12], an increase in the number of freeze-thaw cycles [13,14], and a high speed of freezing and thawing processes [1] can have negative effects on cells. However, the question of how the long-term cryopreservation affects cells remains open. In this regard, this study was aimed to conduct a comparative cytogenetic analysis of cells of different biological origin and genetic status that have undergone a long-term cryopreservation procedure. In the present study, we analyzed the karyotype of transformed Chinese hamster fibroblasts (CHL V-79 RJK, routinely used for in vitro cytogenetic assays to assess the impact of various agents on mammalian cells), as well as the karyotype and physiology of mesenchymal stem/stromal cells isolated from desquamated human endometrium (eMSCs, a source of non-invasive-derived MSCs suitable for cell therapy), that experience 10-year cryopreservation.

## 2. Results

**Figure 1:**
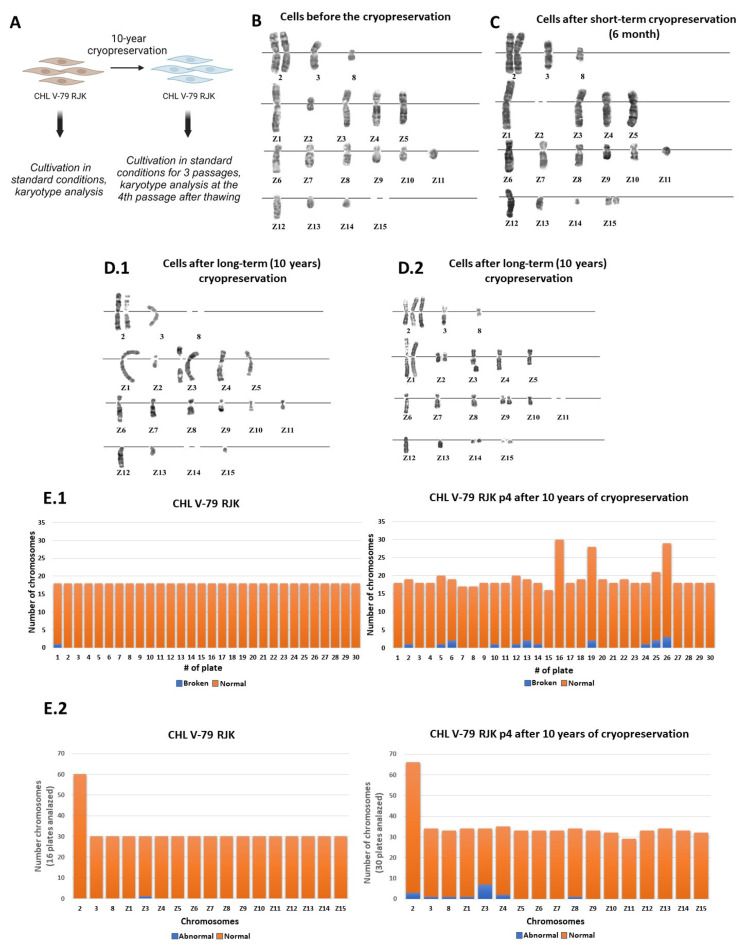
Prolonged cryopreservation causes genomic instability of CHL V-79 RJK cells. (**A**)—Outline of the experiment (Created with BioRender.com.). (**B**)—Typical karyotype of Chinese hamster cells of the CHL V-79 RJK line, before the cryopreservation procedure (*n* = 18). Chromosomes 2, 3, 8 are not rearranged; Z1—Z15—line markers. Chromosome Z15 is absent. (**C**)—The representative karyotype of Chinese hamster cells of the CHL V-79 RJK line, after short-term cryopreservation (6 months) (*n* = 19). Chromosome Z2 is absent. (**D**)—Karyotype of Chinese hamster cells of the CHL V-79 RJK line, subjected to long-term (10 years) cryopreservation. (**D.1**)—Two copies of chromosome Z3 (in one of the copies there is pericentromeric breakage with preservation of p and q—arms), absence of chromosomes 8 and Z14 (*n* = 18). (**D.2**)—Additional copies of chromosomes 2, Z1, Z2, Z9, Z14, Z15; breakage in the terminal part of the q-arm with preservation of chromosomal material in Z3, terminal deletion in one of the copies of Z2; different condensation of homologs of chromosomes 2 and Z1; absence of chromosome Z11 (*n* = 24). (**E**)—Histogram of karyotypic deviations in the cells without cryopreservation and after long-term cryopreservation (10 years). (**E.1**)—on the abscissa axis—the number of the metaphase plates, on the ordinate axis—the number of chromosomes in the analyzed metaphase plate. (**E.2**)—on the abscissa axis—the chromosome number, on the ordinate axis—the number of chromosomes from the analyzed metaphase plates (control cells—30 metaphase plates were analyzed; cells after long-term cryopreservation—30 metaphase plates were analyzed). Blue represents broken chromosomes, orange—normal chromosomes. It is worth noting that chromosome 2 is normally present in the karyotype as two homologs, which is reflected in the histograms.

In the first stage of this work, we analyzed the effect of long-term cryopreservation on the stability of the karyotype of immortalized cells. The CHL V-79 RJK line was used as a model (Figure 1A). This cell line was derived from selection of V-79 cells using 5-bromodeoxyuridine and ouabain treatment to derive a multi-drug-resistant phenotype. The cell line was kindly provided to us by Dr. Ruddle [15]. Since then, over the years of work, the cells were constantly subjected to freeze–thaw cycles, with the duration of each cryopreservation period not exceeding six months. The line was characterized by a stable karyotype that did not change during cultivation including short-term cryopreservation periods [15]. That is why this line was chosen as a model of immortalized transformed cells.

### 2.1. The Effect of Long-Term Cryopreservation on the Karyotype of CHL V-79 RJK Cells

To start with, we carried out karyological analysis of the mitotic chromosomes of the CHL V-79 RJK cells right before cryopreservation. Under standard cultivation conditions, the CHL V-79 RJK karyotype has 18–19 chromosomes. The karyotype was characterized by the presence of three non-rearranged chromosomes 2, 3, and 8, typical for normal Chinese hamster cells, and by the presence of fifteen rearranged CHL V-79 RJK marker chromosomes (Z1–Z15). Each chromosome of a set is usually represented by one copy. The exception is chromosome 2, which has two copies, and chromosome Z2, which may be subject to breakage or elimination. Also, two small chromosomes (Z14–Z15) may be absent in the karyotype, in addition to having two copies. When cultured, the cells maintained karyotypic stability (Figure 1B). Karyotypic analysis of the cells after the short-term cryopreservation for 3 and 6 months did not reveal any significant deviations from their normal karyotype (Figure 1C).

The study of the cell karyotype after long-term storage in liquid nitrogen for 10 years (Figure 1A) showed that upon the culturing of the thawed cells at passage 4, they were characterized by karyotypic instability in half the cases (Figure 1D,E). The identified changes were associated with the presence of additional chromosomal copies or the absence of one or another chromosome, as well as with the detection of pericentromeric or subterminal chromosome breaks (Figure 1D,E). In the Z3 chromosome of the set, the breakage was observed repeatedly (Figure 1E), although the loci of increased fragility were different. The obtained data allow us to conclude that the long-term (10 years) cryopreservation of the CHL V-79 RJK, in contrast to short-term cryopreservation, led to genetic destabilization of their genome.

### 2.2. The Effect of Long-Term Cryopreservation on the Karyotype of Human Endometrial Mesenchymal Stem/Stromal Cells (eMSCs)

Having discovered the effect of long-term cryopreservation on the karyotypic stability of the Chinese hamster fibroblast cell line, a question arose about the effects of long-term cryopreservation on non-immortalized human somatic cells. We chose the eMSCs, line 2304, since MSCs are currently regarded as the most common and valuable substrate for cell therapy. Thus, the genetic stability of these cells is an important subject to study and monitor within the fields of biomedicine and regenerative biology.

**Figure 2:**
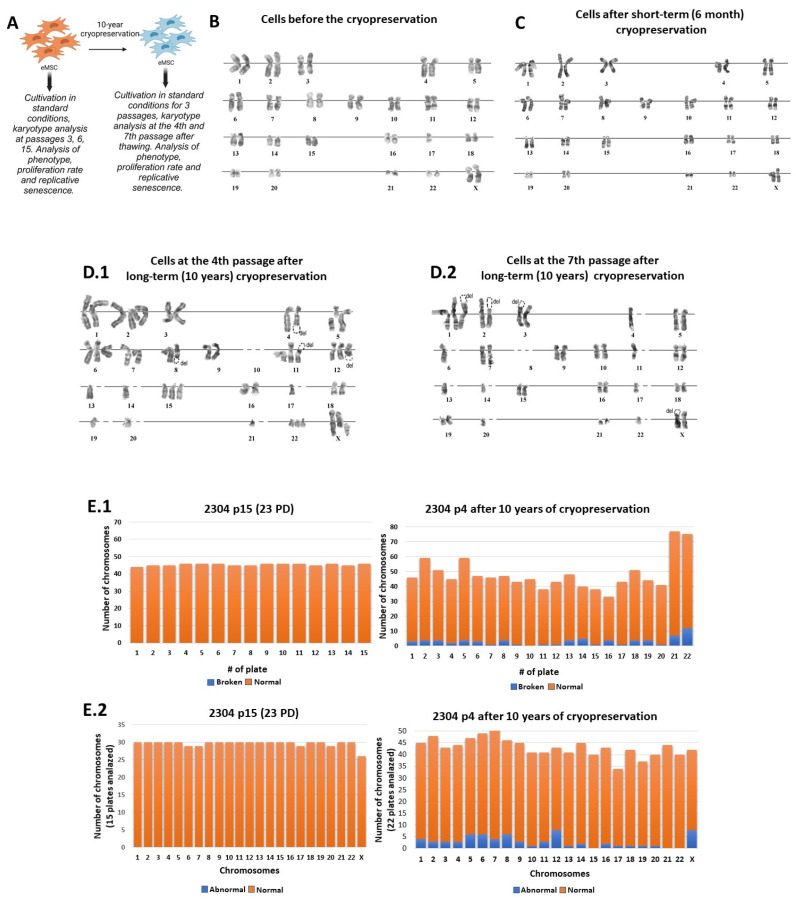
Prolonged cryopreservation causes genomic instability of human eMSCs, line 2304. (**A**)—Outline of the experiment (created with BioRender.com). (**B**)—Karyotype of human eMSCs, line 2304 at passage 15 (23 PD), normal karyotype. (**C**)—The representative karyotype of human eMSCs, line 2304 at passage 15 (23 PD), after short-term cryopreservation (6 months) (*n* = 46). (**D.1**)—Karyotype of the human eMSCs, line 2304 at the fourth passage (12 PD) after long-term (10 years) cryopreservation. Nulesomy of chromosome 10, monosomy 13, 14, 17, 19, 20, 21 chromosomes; trisomy of chromosomes 2, 6, 11, 12, 15, 22; breakdowns with preservation of chromosomal material in one of the homologs of chromosomes 5 and X; terminal deletions in chromosomes 4, 8, 11, 12 (*n* = 43); (**D.2**)—Karyotype of human eMSCs, line 2304 at the seventh passage (16 PD) after long-term (10 years) cryopreservation. Nulesomy of chromosome 8, monosomy of chromosomes 4, 6, 11, 13, 14, 17, 20, 22, trisomy of chromosome 1, terminal deletions in one of the homologs of chromosomes 1, 2, 3, X (*n* = 37). E—Histogram of karyotypic abnormalities in cells without cryopreservation and after long-term cryopreservation (10 years). (**E.1**)—The abscissa axis is the number of the metaphase plate; the ordinate axis is the number of chromosomes in the analyzed metaphase plate. (**E.2**)—on the abscissa axis—the chromosome number, on the ordinate axis—the number of chromosomes from the analyzed metaphase plates (control cells—15 metaphase plates were analyzed; cells after long-term cryopreservation—22 metaphase plates were analyzed). Blue represents broken chromosomes, orange—normal chromosomes.

We analyzed the karyotyping stability of eMSCs, line 2304 at different stages of cultivation (passages 3, 6, 15 with corresponding population doublings (PD) = 5, 9, 23), and the data allowed us to consider it as genetically stable (Figure 2A,B) [16]. There were some rare karyotypic defects in the early passages of cultivation such as a violation of chromosome copy numbers, differences in condensation between homologs, and inter-chromosomal associations. By passage 15 (23 PD) after the derivation, the line was considered as karyotypically stable (Figure 2A,B). The routine short-term freezing/thawing of cellular material did not lead to significant changes of the karyotype (Figure 2C) [16].

The derived eMSCs, line 2304 was cryopreserved at 6 PD and stored in liquid nitrogen for 10 years (Figure 2A). Upon thawing after the long-term cryopreservation, the cells were cultured under the standard conditions. The thawed eMSCs were karyotyped at passage 4 (12 PD) of culturing. The G-banded metaphase chromosome analysis showed that the genetic stability of the thawed eMSCs after long-term cryopreservation, as well as in CHL V-79 RJK cells after many years of cryopreservation, was impaired. 

After 10 years of cryopreservation, the thawed eMSCs were characterized by the presence of aneuploidy, chromosomal aberrations, impaired condensation in homologs, and inter-chromosomal associations (Figure 2D). Aneuploidy was present in all karyotyped cells, but possessed different manifestations from cell to cell. In some cases, the violation of copy number was observed in only one of the chromosomes of the set, while in other cells, several chromosomes of the set were affected.

We observed that the variability in the number of chromosomal copies, both within a cell and from cell to cell, diverged from 0 to 3 (Figure 2E). In the population, the near-diploid cells prevailed. The presence of chromosomal aberrations was associated with increased fragility of the chromosome set. Failures occurred in the pericentromeric and terminal regions of the chromosomes, with preservation or deletion of the genetic material.

The preservation of genetic material in the pericentromeric regions was accompanied by the appearance of two new chromosomes instead of one. Some chromosomes (most often chromosomes 12 and X) were characterized by increased fragility (Figure 2E). Discrepancies in the condensation between homologs, as well as inter-chromosomal associations were rare.

During the following short-term (three passages) cultivation at 18 PD of thawed eMSCs, we observed that the karyotypic heterogeneity contributed to the emergence of genetically diverse defective cells. Some single cells were near-triploid and near-tetraploid. In some cases, both homologous chromosomes were fractured. At the same time, we found that the chromosomes’ fragility pattern was different from passage to passage, and that there were no permanent genetic mutations or aberrations in the thawed eMSCs culture. 

### 2.3. Phenotypic and Functional Characteristics of Thawed eMSCs Subjected to Long-Term Cryopreservation

Since we discovered an increased fragility of chromosomes in the thawed eMSCs after long-term cryopreservation, it was of interest to analyze their phenotypic characteristics.

**Figure 3:**
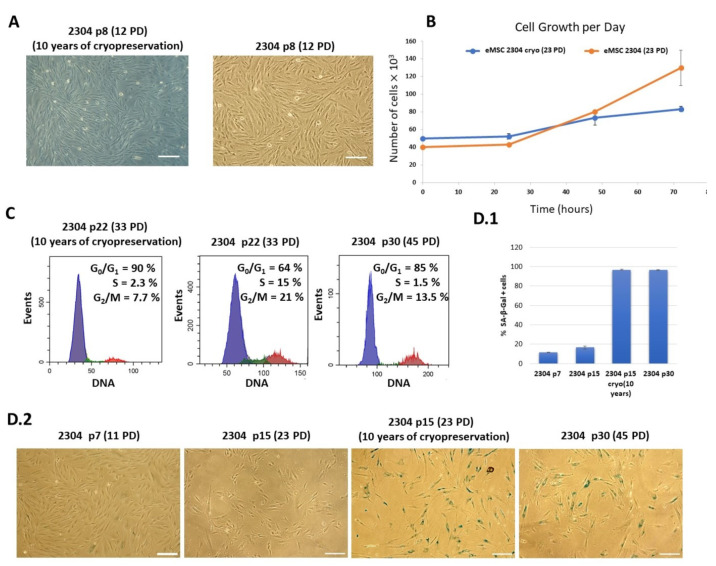
Prolonged cryopreservation leads to the early manifestation of cellular senescence features in human eMSCs, line 2304. (**A**)—The similar morphology of cryopreserved (left panel) and continuous eMSC culture (right panel), scale bar = 200 μm. (**B**)—Growth curves of the continuous and the thawed eMSCs, line 2304 at p15 (23 PD). Data are shown as mean ± SD (N > 3). (**C**)—Proliferation arrest of the thawed (after 10 years cryopreservation) eMSCs, line 2304 on p22 (33 PD) and the continuous eMSC cultures on p22 (33 PD) and p30 (45PD) (N = 3). The flow cytometry analysis was performed in 48 h after seeding. Different colors mark different phases of the cell cycle: blue—G_0_/G_1_ phase, green—S phase, red—G_2_/M phase. (**D**)—Expression of SA-β-gal (quantification (**D.1**) and images of the SA-β-gal (**D.2**)) Data are shown as mean ± SD (N > 3). SA-β-Gal staining: the continuous eMSCs at early 7 (12 PD) and midlife 15 (23 PD) passages, the thawed (after 10 years of cryopreservation) eMSCs at the average passage 15 (23 PD), and the continuous eMSC culture at the late 30th passage (45 PD), scale bar = 200 μm.

Our study showed that the thawed eMSC lines retain their morphology upon culturing (Figure 3A). Further, by means of flow cytometry, we analyzed the phenotypic expression of surface CD markers of the thawed cells. It was found that the profile of CD markers after long-term cryopreservation remained unchanged, and in accordance with the criteria established by the International Society for Cellular Therapy. The thawed eMSCs expressed surface markers CD13, CD44, CD73, CD90, and CD105 and did not express hematopoietic markers CD34, CD45, and HLA-DR (class II). The expression profile of the CD markers did not change during the subsequent cell cultivation until their replicative senescence (Table 1).

At the same time, we observed the significant distinction in the functional status of the cryopreserved eMSCs. Upon subevent culturing of thawed eMSCs, we revealed the substantial decrease in the proliferation rate of continuously cultured eMSCs and eMSCs after long-term cryopreservation based on growth curve measurements (Figure 3B). At the same stage of 23 PD cultivation, their population doubling time differed by 1.5-fold to an average of 40.8 h, instead of 22–23 h for the same continuous eMSC culture (Figure 3B). Further, our study showed that long-term cryopreservation induced the features of cellular senescence of the thawed cultured eMSCs at 28 PD versus the average 45 PD for the same continuous cell culture. The flow cytometry analysis of the cell cycle phase distribution revealed that most of the thawed eMSC population at 28 PD was accumulated in the G0/G1 phase (Figure 3C) and only around 3% of the cells were in the S phase. The continuous eMSCs, line 2304 culture showed a similar cell cycle distribution at an average of 45 PD (Figure 3C). These results were confirmed by an increase in senescence-associated β-galactosidase (SA-β-gal) activity in the thawed eMSC culture. As shown in Figure 3D1, the number of SA-β-gal positive cells in eMSCs without long-term cryopreservation was 17 ± 3% while in eMSCs after 10 years of cryopreservation at the corresponding passage (passage 15), it was strikingly higher—97 ± 2%. Such a high level of SA-β-gal positive cells corresponds to replicatively aged non-frozen cells at the 30th passage of cultivation (Figure 3D).

Similar results were obtained for another eMSC 2804 cell line that was derived from a different donor [17] and subjected to 10 years of cryopreservation as well. The thawed eMSCs 2804 retained their morphology and phenotype during culturing. However, similarly to eMSCs, line 2304, the thawed cells demonstrated a decrease in proliferative rate and a manifestation of the cellular senescence features—enlarged cell size, increase in SA-β-gal activity, proliferation arrest—at significantly earlier passages/PD than the continuous eMSCs 2804 (Figure S1).

Thus, we can conclude that long-term cryopreservation (10 years) results in a decrease in proliferative activity and the induction of the features of premature senescence in eMSC cultures.

## 3. Discussion

Cryopreservation is a necessary and essential step for the preparation and use of cells in clinical studies to accumulate large numbers of cells that have undergone testing and that are ready at any time for administration to patients. In this regard, the impact of cryopreservation on therapeutic potential and karyotypical stability of cultured cells remains a focus of intense research. A number of studies have shown that multiple freeze–thaw cycles can lead to a decrease in the proliferative properties and regenerative potential of cells [14,18]. At the same time, few studies have been devoted to the effects of long-term cryopreservation on cells and biomaterials. In this study, we addressed this issue. 

The first part of the work was devoted to studying the effect of cryopreservation on the karyotype of immortalized cells. The results of karyological analysis showed that the transformed cells of the CHL V-79 RJK line, used in this work as a model system, retained a stable karyotype during relatively short storage in liquid nitrogen (3 and 6 months). The long-term (10 years) cryopreservation of the CHL V-79 RJK cells led to the destabilization of their genome. We observed a change in the copy number of chromosomes, an increasing number of chromosomes involved in rearrangements, an elevated fragility of chromosomes that led to their breakdowns, impaired chromosome condensation, and chromosomal adhesions (ectopic conjugation). The repeated breakdowns in different localizations of the Z3 chromosome were evaluated as non-random, caused by prolonged exposure of the cells to ultra-low temperature conditions.

According to the literature, even a single stable change in cell karyotype leads to disruption of the mechanism of cell division and destabilization of the cell genome. The karyotypic instability of cell cultures undergoes two stages: the first stage is nonclonal, and associated with the accumulation of random karyotypic changes in the population, which can disappear at any passage of culturing; the second stage of karyotypic instability is clonal, with certain chromosomal changes (aneuploidy or chromosomal aberrations) becoming permanent and resulting in a modified cell karyotype [19]. In the case of CHL V-79 RJK cells, we observed an increased genetic instability of chromosome Z3. Therefore, we cannot exclude the possibility that the cells with the structurally altered chromosome, Z3, may acquire a selective advantage of a clonal nature upon culturing. 

We identified another destabilization of the cellular genome of CHL V-79 RJK cells after 10 years of cryopreservation. This destabilization is associated with an increase in the number of copies or the loss of individual chromosomes of the karyotypic set. It is considered that this is due to a disruption in the mechanism of cell division. Currently, a failure in the cell division program is considered as one of the mechanisms of destabilization of the cellular genome, and may lead to the transition of cells to a malignant state [20].

McGranahan et al. (2012) showed that in human cells, the presence of even one additional chromosomal copy in the karyotype causes genomic instability and the disruption of DNA replication [21]. The dynamics of continuous sequential changes in the structure of the genome, growing over time in a “snowball effect”, can lead to a significant reorganization of the cellular genome, referred to as “karyotypic chaos”. Signs of “karyotypic chaos”, along with changes in the number of chromosomes and chromosomal/chromatid breaks, include varying degrees of chromosome condensation and inter-chromosomal adhesions [22]. We observed all these impairments in CHL V-79 RJK cells that had undergone 10 years of cryopreservation. In sum, the data obtained indicate that changes in the CHL V-79 RJK karyotype after long-term cryopreservation may lead to a more malignant cell phenotype. 

The second part of the study investigated how long-term cryopreservation affects non-immortalized human cells using eMSC cell lines as the model. At present, human MSCs are widely used in clinical trials due to their ease of derivation and cultivation, as well as their paracrine therapeutic properties. By mid-2019, in the United States alone, MSCs were involved in 368 trials for the treatment of diseases such as stroke, coronary heart disease, multiple sclerosis, and arthritis (ClinicalTrials.gov) [1]. However, few studies have been devoted to the problem of long-term cryopreservation of MSCs, and there are no data on the long-term freezing of eMSCs. eMSCs from desquamated endometrium have great potential in regenerative medicine due to the non-invasive procedure for their derivation and their high proliferative rate ex vivo (about 45 PD in culture). This proliferative rate significantly exceeds the perioperative potential of MSCs from bone marrow and umbilical cord blood [23,24].

In the present study, after the long-term cryopreservation of eMSCs, we revealed the karyotypic abnormalities, similar to those that we have observed in the Chinese hamster cells CHL V-79 RJK. Among the karyotypic changes were aneuploidization of the chromosome set, an increased fragility of chromosomes, and a violation of compactization in homologs. Unlike CHL V-79 RJK, almost all chromosomes (most frequently chromosomes 12 and X) of the set were involved in the process of destabilization of the cellular genome of the eMSCs. The chromosomal damage affecting pericentromeric regions was in some cases accompanied by the preservation of genetic material in the form of independent chromosomes. The non-centromere material was eliminated in most cases during culturing. Analysis performed after the subsequent short-term cultivation of eMSCs showed that the genetic defects were not clonal, but the fragility of chromosomes persisted with further passaging of thawed cells. Importantly, before long-term cryopreservation, the derived eMSC line was found to be karyotypically stable.

We performed characterization of thawed eMSC and found that the phenotype and morphology of cells subjected to 10 years of cryopreservation did not change during the subsequent culturing till their replicative senescence. The eMSC lines had positive expression of CD73, CD90, CD105, CD13, and CD44 markers, and the absence of expression of surface markers CD34, CD45, and HLA-DR (class II). Morphologically, the eMSCs retained their adhesiveness to plastic, a fibroblast-like shape, and formed a monolayer. This correlates with the findings of other researchers on the phenotypic stability of various types of MSCs after cryopreservation [1,9]. At the same time, when comparing the proliferative potential of thawed and continuous eMSCs, we found that long-term cryopreservation caused serious disturbance in cell proliferation activity and accelerated the manifestation of the signs of cellular senescence. The number of PD achieved prior to the proliferation arrest was 28 vs. 45 reached by the continuous cell culture. 

In general, it is known that the shortening of chromosomes in the terminal part leads to the senescence of cultivated cells. The terminal part of chromosomes (telomeres) are guanine-rich tandem repeats of DNA. They ensure chromosome structural stability during cell division. In each cycle of cell division, the length of telomeres decreases, which ultimately leads to the aging of non-transformed cells [25]. Thus, it can be assumed that the telomere damage (and other accumulated defects in the genome) of the eMSCs, which we have found by karyotyping after the long-term cryo-freezing of cells, is the path to their premature senescence. Interestingly, in thawed cells, we observed the karyotypic changes similar to those which we had found previously in the same eMSC line when the cells were subjected to X-ray irradiation or heat shock [26]. Therefore, it suggests that long-term cryopreservation can be considered as a severe stress factor for these cells. 

Discussing the significance of our results, it is worth noting, however, that the cell culture cryopreservation facility used in this work was not certified for medical applications. Consequently, we cannot unequivocally state that long-term freezing of cells in medical institutions will lead to similar results. Nevertheless, considering that MSC banking accompanied by long-term storage of cells in liquid nitrogen is a significant direction for the development of regenerative medicine in general, the effects found in the present study (increased chromosome fragility and premature aging of long-term frozen MSCs) require further and more detailed study.

## 4. Materials and Methods

Cell Cultures. In this work, we used three cell lines of different biological origin and genetic status. The immortalized transformed Chinese hamster fibroblast line, CHL V-79 RJK, was provided by Dr. F. Ruddle, Yale University, New Haven, CT, USA. Due to the fact that it is an immortalized cell line, we counted the passages from the beginning each time the cells were thawed. The duration of each cryopreservation before the experiment was no more than 6 months. Two lines of human endometrial mesenchymal stem/stromal cells (eMSC 2304 and eMSC 2804) were isolated from the desquamated endometrium of the menstrual blood of healthy donors. The eMSC lines used in this work were kindly provided by the Cell Culture Bank of the Institute of Cytology RAS at early passage, with the duration of cell cryopreservation not exceeding 6 months [17]. These eMSC lines are non-immortalized cell lines and passages were calculated at each passage from the moment the cell line was obtained. After obtaining the cell line, a portion of the cells in the earliest passages without cryopreservation were transferred to us for research. In this regard, we had an opportunity to analyze cells in the earliest passages (at the 3rd passage) without cryopreservation. In further work, cells were subjected to short-term cryopreservation (no more than 6 months). The cells were cultivated in T25 flasks (Fisher Scientific, Waltham, MA, USA) in DMEM/F12 growth medium (Gibco, Grand Island, NY, USA) containing 10% fetal bovine serum (FBS, HyClone, Logan, UT, USA), 1% L-glutamine (Gibco, USA), and 1% penicillin–streptomycin (Gibco, USA). All cell cultures were maintained at 37 °C in a humidified chamber with 5% CO_2_ and subcultured at a 1:3 ratio twice a week. The number of cell population doublings was calculated using the formula:

PD = 1.5 × Np(1)

where Np = number of cell passage cycles.

Cryopreservation. Cells were detached from the plastic surface with a 0.05% trypsin-EDTA solution (Gibco, USA), and centrifuged in the culture medium at 1500 rpm for 5 min. The supernatant was removed, and the cells was resuspended in a solution of 90% bovine serum (HyClone, USA) and 10% DMSO (Sigma, Burlington, MA, USA). Resuspended cells were transferred into cryovials (Nunc, Rochester, NY, USA) and frozen at a rate of 1 °C/min, followed by storage in liquid nitrogen (−196 °C). When thawing, the cryovials with cells were quickly heated in a water bath at 37 °C. Then, the cells were transferred to a centrifuge tube, the culture medium was added, and the cells were centrifuged at 1500 rpm for 5 min. The cells were removed (washed) from DMSO, and placed into culture flasks for further cultivation in accordance with the standard protocol.

Karyotyping. To prepare metaphase chromosomes, cells were seeded at a density of 14–15 × 10^3^ cells/cm^2^. After 24–25 h, the mitostatic agent, colchicine (Merk, Rahway, NJ, USA), was added to the cell culture dish at a final concentration of 3.6 μg/mL for CHLV-79 RJK, or 0.02 mg/mL of colcemid (Sigma, USA) for the eMSCs. After 1 h of incubation, the medium was removed, and the cells were washed with PBS solution (Sigma, USA) and enzymatically detached from the plastic with a 0.05% trypsin/EDTA solution.

The cell suspension was centrifuged, the supernatant was removed, and the sediment was resuspended and subjected to hypotonic treatment with a 0.56% KCl solution (Reakhim, Moscow, Russia). After hypotonic treatment, the cell suspension was centrifuged (1000 rpm for the CHL and 1500 rpm for the eMSCs), the supernatant was removed, and the cells were fixed for 1.5 h with a cold mixture of methanol and acetic acid (3:1). The fixed material was placed onto cold, damp glass slides. The slides were then dried at room temperature for 1 week. Metaphase plates were stained with Giemsa stain (BDH, London, England) in PBS after preliminary trypsinization. Karyotypic analysis was carried out using an Axio Scop light microscope (Carl Zeiss, Oberkochen, Baden-Württemberg, Germany) at objective magnifications of 20×, 40×, 100×. Chinese hamster chromosomes were identified according to nomenclature [27] and human chromosomes were identified according to international nomenclature [28].

Determination of senescence-associated β-galactosidase (SA-β-Gal) activity. Cells were seeded into Petri dishes with a diameter of 3 cm and cultured for 3 days. Then the medium was removed, the cells were washed with PBS, fixed with 4% formaldehyde, and stained with the SA-β-Gal kit (Cell Signaling, Danvers, MA, USA) according to the manufacturer’s instructions. SA-β-Gal activity was determined by blue staining in the cells when viewed under a light microscope. The quantification was performed using ImageJ software (the version number 1.53t from 24 August 2022). At least 150 cells per experiment were counted in the analysis. 

The senescent cells were identified by blue color in their cytoplasm.

Cell growth kinetics. Cell growth properties were assayed by measuring growth curves using flow cytometry (Beckman Coulter, Chaska, MN, USA). In 3.5 cm Petri dishes, 5 × 10^5^ eMSC cells per plate were seeded. The number of cells was calculated by means of flow cytometry for three consecutive days. Three plates were used for each measurement for calculation of the mean value. Population doubling time was calculated according to the formula:

td = (2*∆t*x_0)/x(2)

where td = the doubling time, x_0 = the initial cell number, x = the number of cells at the end of the current time interval, and ∆t = the time interval. 

Immunophenotyping. Immunophenotypic analysis of the obtained eMSCs for CD surface markers was carried out using an Epics XL flow cytometer (Beckman Coulter, USA). A single cell suspension was prepared using 0.05% trypsin and EDTA solution. The cells (1 million/mL) were resuspended in a PBS solution containing 5% FBS. Antibodies conjugated to FITC or phycoerythrin were used for analysis: CD13, CD34, CD44, CD45, CD73, CD90, CD105, and HLA-DR.

Cell cycle analysis was performed using flow cytometry. The cells were detached using 0.05% trypsin/EDTA solution (pelleted by centrifugation and washed with phosphate-buffered saline (PBS)). One part of each sample was used for propidium iodide (PI; 50 μg/mL) staining and subsequent cell viability analysis. Another part of each sample was used to analyze the cell cycle phase distribution. The cells were incubated for 30 min in 300 μL of PBS containing 200 μg/mL of saponin, 250 μg/mL RNase, and 50 μg/mL PI at room temperature and analyzed using a Coulter EPICS XL Flow Cytometer (Beckman Coulter). The cell cycle distribution was analyzed using the WinMDI program.

Statistics. All experiments were repeated at least 3 times. Data are presented as means ± SD, when indicated. Statistical significance in the comparison of cell growth curves was evaluated by applying the Compare Groups of Growth Curves test as described in [29], and *p* < 0.05 was considered to be significant. Microscopy images and flow cytometry histograms shown correspond to the most representative experiments.

## 5. Conclusions

The long-term cryopreservation of cells of various types and biological origins can lead to irreversible changes in their karyotypes. In eMSC cultures, this can facilitate premature cellular senescence. In order to avoid the risks arising from the long-term cryopreservation of eMSCs that are used for biomedicine purposes, the monitoring of their cytogenetic characteristics is necessary.

## Figures and Tables

**Table 1:** Immunophenotyping of eMSCs, line 2304 (12 PD) before cryopreservation and eMSCs, line 2304 (17 PD) after long-term cryopreservation-specific CD markers.

Cell Surface Markers	Percent of Cells Expressing Marker (%)	
	Continuous Culture	Cryopreserved Culture
	MSC 2304 (12 PD)	MSC 2304 (17 PD)
CD13	99.1 ± 1	99.7 ± 1
CD44	99.6 ± 2	99.9 ± 1
CD73	99.7 ± 0.3	96.2 ± 2.4
CD90	95.2 ± 2	98.4 ± 1
CD105	98.2 ± 3	83.1 ± 4
CD34	0.37 ± 1	1.13 ± 0.4
CD45	2.40 ± 3	7.10 ± 2
HLA-DR (class II)	0.08 ± 1	0.56 ± 1

## Data Availability

Data are contained within the article and Supplementary Materials.

## Supplementary Materials

The following supporting information can be downloaded at: https://www.mdpi.com/article/10.3390/ijms25031467/s1.
