# Supplementary material for: Long-Term Cryopreservation May Cause Genomic Instability and the Premature Senescence of Cells
Source: Int J Mol Sci. 2024 Jan 25;25(3):1467. doi: 10.3390/ijms25031467 (PMC10855830; doi:10.3390/ijms25031467)
Supplement: Supplementary file 1 [file ijms-25-01467-s001.zip › ijms-2776829-supplementary.pdf]

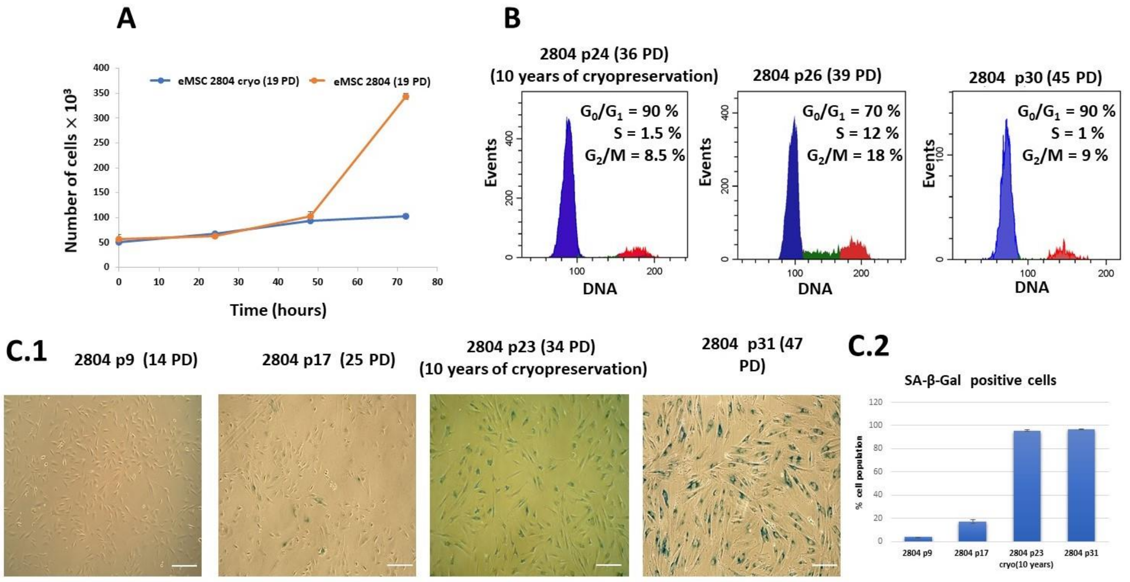

Figure S1. Prolonged cryopreservation leads to the early manifestation of cellular senescence features in human eMSCs, line 2804. A – Growth curves of the thawed (after 10 years cryopreservation) eMSCs, line 2804 at middle passages p13 (19 PD) and the continuous eMSCs, line 2804 p13 (19 PD). Data are shown as mean ± SD (N > 3). B - Proliferation arrest of the thawed (after 10 years cryopreservation) eMSCs 2804 at the p26 (39 PD) (N=3). The flow cytometry analysis was performed in 48 hours after seeding. C - Expression of SA-β-gal (images of the SA-β-gal (C.1) and quantification (C.2)) Data are shown as mean ± SD (N > 3). SA-β-Gal staining: the continuous eMSCs, line 2804 at early passage 14 PD, and mid-life passage of 23 PD; the thawed (after 10 years of cryopreservation) eMSCs, line 2804 underwent replicative senescence at 34 PD, and the continuous eMSCs line 2804 underwent replicative senescence at 47 PD, scale bar=200 μm.
